# Cervical Spinal Cord Injury Without Radiographic Evidence of Trauma (SCIWORET) in an Adult: A Rare Diagnosis in the Intensive Care Unit

**DOI:** 10.7759/cureus.110400

**Published:** 2026-06-07

**Authors:** Joana Vaz, Nuno Catorze, Jorge Nunes, José Ramalho, Marta Sousa

**Affiliations:** 1 Critical Care Medicine, Unidade Local de Saúde (ULS) do Médio Tejo, Abrantes, PRT; 2 Intensive Care Unit, Unidade Local de Saúde (ULS) do Médio Tejo, Abrantes, PRT; 3 Intensive Care Unit, Hospital da Luz Lisboa, Lisbon, PRT

**Keywords:** cervical spondylosis, hyponatremia, sciworet, spinal cord infarction, spinal cord injury

## Abstract

Spinal cord injury without evidence of trauma (SCIWORET) is an uncommon clinical entity in adults, typically associated with pre-existing degenerative spinal conditions, as well as vascular susceptibility. Its diagnosis in the intensive care unit (ICU) is challenging due to the confounding effects of sedation and post-arrest encephalopathy. A 67-year-old man suffered a cardiac arrest triggered by severe hyponatremia (Na+ 115 mmol/L ) during colonoscopy preparation. After resuscitation, he exhibited spastic tetraparesis. While CT was negative for acute trauma, MRI revealed a hyperintense T2-signal from the bulbomedullary junction to C3, with a characteristic "H-sign" in the anterior horns, confirming spinal cord infarction or transverse myelitis of undetermined etiology, favoring the diagnosis of SCIWORET. Following intensive rehabilitation, the patient achieved near-total functional recovery. SCIWORET should be suspected in post-arrest patients with "neurological mismatch." Early MRI is essential to differentiate focal spinal ischemia from global hypoxic brain injury, facilitating early multidisciplinary rehabilitation.

## Introduction

Cervical spondylosis is a ubiquitous degenerative condition, affecting approximately 75-95% of individuals over the age of 65 [1]. While often asymptomatic, it serves as a critical predisposing factor for spinal cord injuries, even in the absence of high-energy trauma. A specific and frequently underdiagnosed clinical entity is spinal cord injury without evidence of trauma (SCIWORET), which accounts for 5-16% of all spinal cord injuries in trauma and general spinal cord injury series [1,2]; its exact incidence in ICU populations is still unknown.

The term evolved from the pediatric concept of SCIWORA (spinal cord injury without radiologic abnormality), characterized by acute traumatic myelopathy despite normal plain radiographs and CT studies [3]. In children, this is attributed to the inherent elasticity of the juvenile spine. In contrast, SCIWORET in adults is typically anchored in underlying mechanical vulnerabilities, such as cervical spondylosis, ossification of the posterior longitudinal ligament (OPLL), or spinal canal stenosis [2,4]. SCIWORET is the current term of choice for adults presenting with spinal cord dysfunction in the absence of acute traumatic injury, even when degenerative radiographic changes, such as spondylosis or canal stenosis, are present, distinguishing it from the pediatric SCIWORA concept, where the spine is radiographically normal.

The diagnostic landscape was transformed by the advent of magnetic resonance imaging (MRI), which can detect intramedullary lesions in up to 90% of symptomatic patients who present with unremarkable CT scans. In the intensive care unit (ICU), recognizing SCIWORET is exceptionally difficult. Neurological deficits are often masked by sedoanalgesia, neuromuscular blockade, or the clinical assumption that motor weakness is solely a component of post-cardiac arrest encephalopathy or critical illness polyneuromyopathy. While mechanical compression remains the most well-recognized precipitant, SCIWORET can also result from ischemic etiologies, including anterior spinal artery infarction, a unique and underdiagnosed mechanism that is central to this case.

We present a rare case of SCIWORET in an adult where metabolic and hemodynamic triggers converged on a chronically compromised cervical spine.

## Case presentation

A 67-year-old man with a significant psychiatric history, including schizophrenia, epilepsy, and depression, was admitted to a psychiatric ward for an acute psychotic episode. During his hospitalization, he underwent bowel preparation for a colonoscopy to investigate potential clozapine-induced ischemic colitis. During the preparation phase, the patient was found collapsed in cardiorespiratory arrest. Advanced life support (ALS) was initiated, and return of spontaneous circulation (ROSC) was achieved after two minutes of resuscitation. Initial laboratory results revealed severe hyponatremia (115-118 mmol/L), mild metabolic acidosis (pH 7.15, lactate 9.7 mmol/L), and mild hypokalemia. ECG showed sinus rhythm without ischemic changes. He was intubated for airway protection and transferred to our emergency department.

On arrival, hemodynamic parameters were stable (blood pressure 151/69 mmHg, heart rate 62 beats per minute, oxygen saturation (SpO₂) 99%), with no signs of trauma. Whole‑body CT, including head and cervical angiography, showed only diffuse cerebral atrophy, mild carotid calcification, and degenerative cervical changes without evidence of fracture or laceration. A chest CT identified right lower‑lobe aspiration pneumonitis.

He was admitted to the ICU for post-cardiac arrest management with targeted temperature control and gradual correction of hyponatremia. Sodium correction was achieved within guideline-recommended limits, rising from 115-118 mmol/L on admission to 125 mmol/L on day 2 and 131 mmol/L on day 3, confirming adherence to the ≤8 mmol/L/day target and minimising the risk of osmotic demyelination syndrome (Table 1).

**Table 1:** Evolution of laboratory and arterial blood gas (ABG) parameters pCO2: partial pressure of carbon dioxide; HCO3-: bicarbonate; Na: sodium; K: potassium

Parameter	Initial Arrest (Ward)	ICU Admission	Reference Range
pH	7.15	7.41	7.35 – 7.45
pCO2 (mmHg)	47	39	35 – 45
HCO3- (mEq/L)	15.3	24.7	22 – 26
Na+ (mmol/L)	115	118	135 – 145
K+ (mmol/L)	3.2	3.2	3.5 – 5.0
Lactate (mmol/L)	9.7	2.6	< 2.0
Troponin (ng/mL)	--	8.9	< 0.04

The elevation of troponin was attributed to type 2 myocardial infarction in the context of cardiac arrest and systemic hypoperfusion. Transthoracic echocardiography showed preserved global systolic function, no regional wall motion abnormalities, and no morphofunctional valvular changes, excluding primary myocardial ischaemia as the cause for the arrest.

A "neurological mismatch" became evident upon the tapering of sedation. While the patient regained consciousness and followed simple commands with cranial nerves, he exhibited spastic tetraparesis with grade 1/5 muscle strength in the lower limbs and upper limb plegia, accompanied by hypesthesia of both upper and lower limbs. Given the patient remained sedated almost 72 hours following resuscitation, the initial flaccid phase of spinal shock wasn't clinically assessable; the first neurological examination was performed upon sedation withdrawal, at which point upper motor neuron signs were already apparent. Cough and swallow reflexes were preserved. Sphincter incontinence was present, and deep tendon reflexes were bilaterally absent. Initial cervical CT showed multilevel disco-osteophytic protrusions but no acute fractures or listhesis.

**Figure 1:**
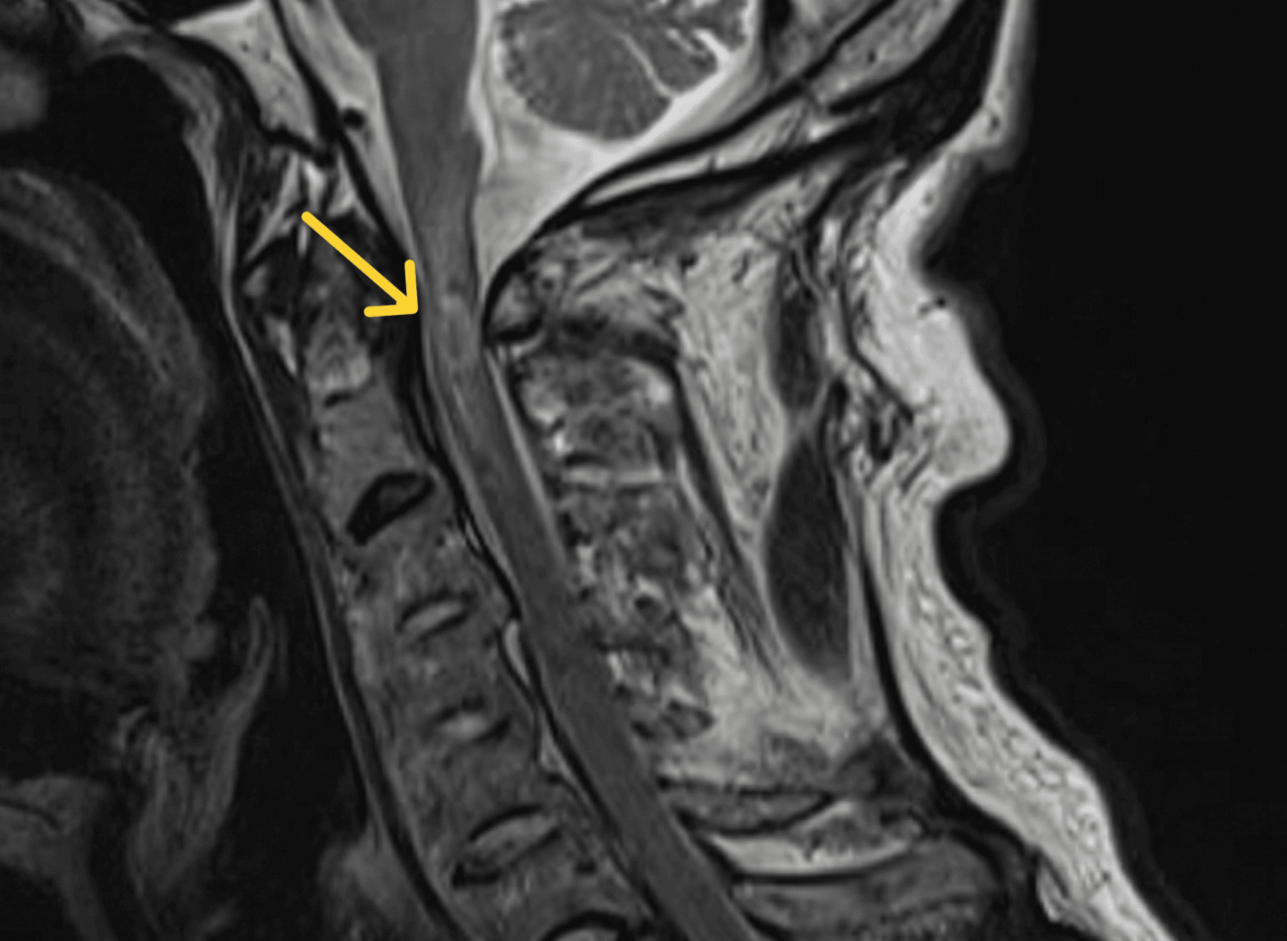
Sagittal T2-weighted MRI of the cervical spine Multilevel spondylotic changes and a diffuse intramedullary T2 hypersignal extending from the bulbomedullary junction to the C3 segment (yellow arrow) can be seen. The most prominent signal changes are observed at the C1-C2 level. Background multilevel disco-osteophytic changes and reduced anterior subarachnoid space are consistent with pre-existing cervical spondylosis.

**Figure 2:**
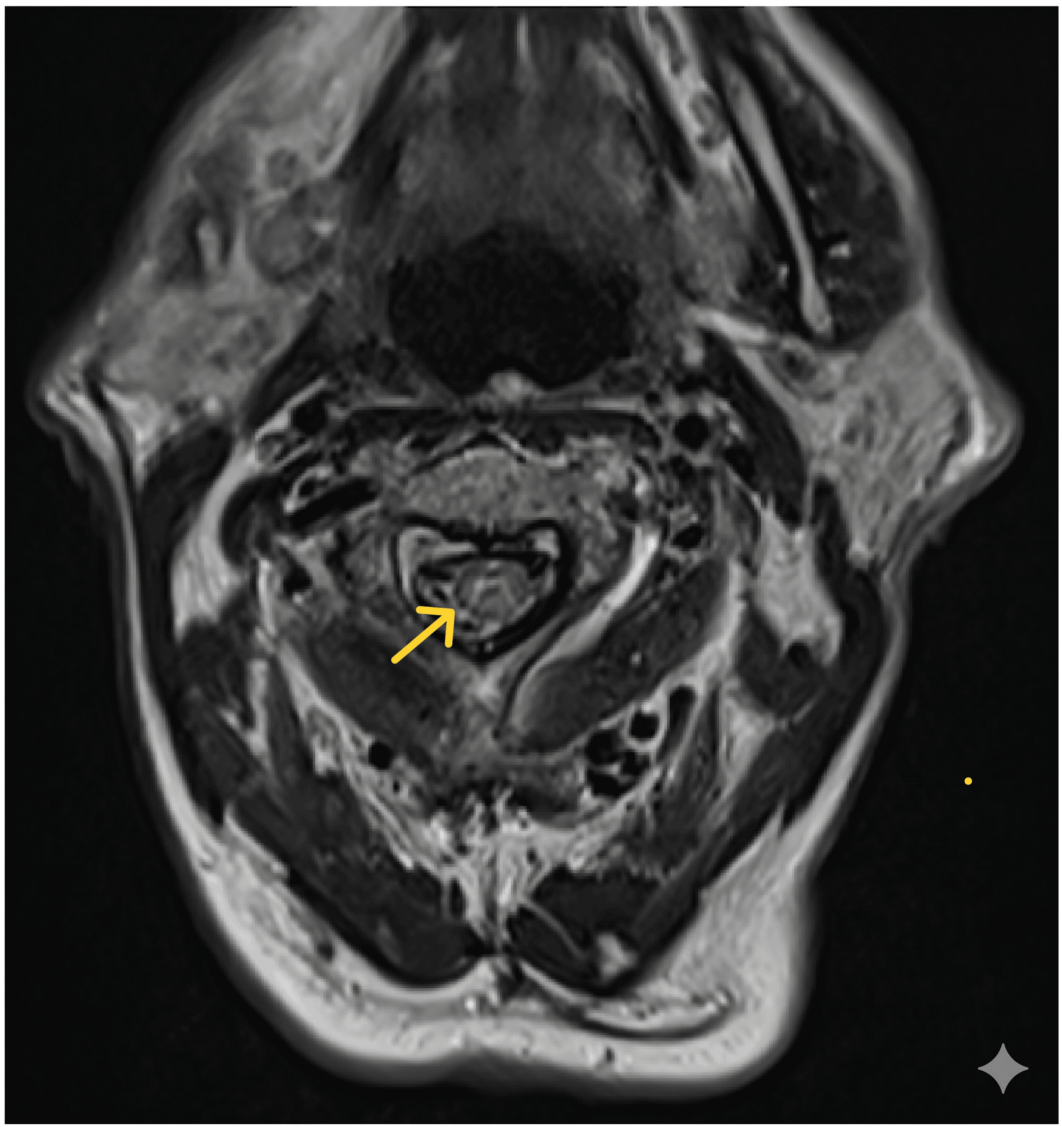
Axial T2-weighted MRI at the C2 level The "H-sign" (yellow arrow) can be seen, characterized by hyperintensity restricted to the gray matter anterior horns, diagnostic of spinal cord infarction. See Figure 3 for anatomical correlation.

**Figure 3:**
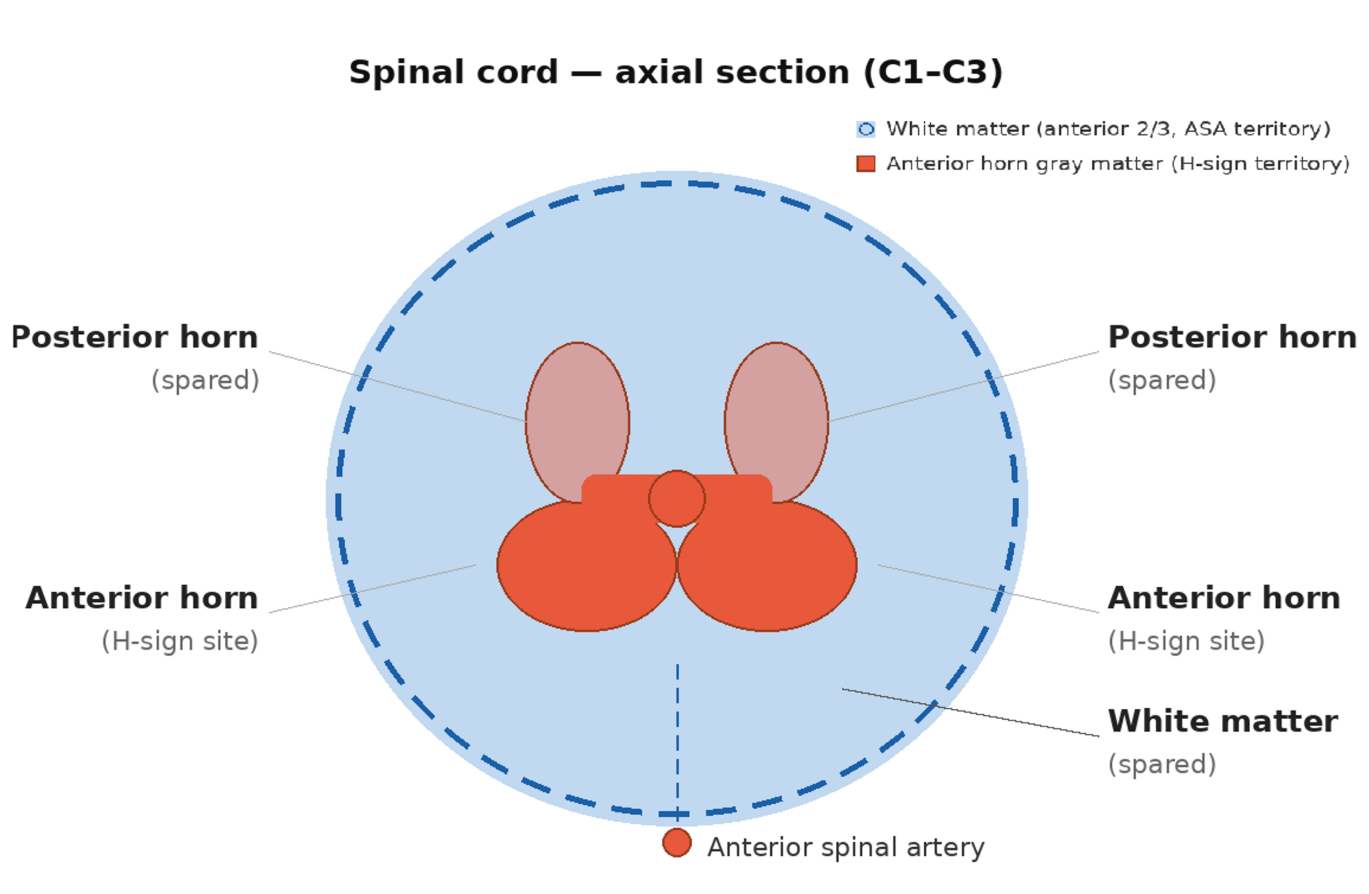
Schematic diagram of a normal spinal cord axial section illustrating the territory of the anterior spinal artery (ASA) The anterior horn gray matter (red) represents the ASA territory and the site of the T2 hypersignal ('H-sign') observed in Figure 2. The posterior horns and white matter, supplied by the posterior spinal arteries and peripheral vasocorona, were radiologically spared. The figure was created for illustrative purposes by the authors using Python (Pillow library; https://python-pillow.github.io/)

Urgent MRI of the cervical spine was performed on the same day that sedation was withdrawn, immediately upon recognition of the neurological deficits, which demonstrated a diffuse area of T2-weighted and short TI inversion recovery (STIR) hypersignal extending from the bulbomedullary junction to the C3 segment. Crucially, axial sequences revealed the "H-sign," where the hypersignal was strictly confined to the gray matter of the anterior horns. This pattern is a radiologic hallmark of spinal cord infarction, specifically within the territory of the anterior spinal artery (Table 2, Figures 1-3). 

**Table 2:** Summary of multi-modal imaging and neurological findings STIR: short TI inversion recovery; RLL: right lower lobe; TAP: thorax, abdomen, and pelvis; SCIWORET: spinal cord injury without evidence of trauma

Modality	Key Positive Findings	Clinical Impression
Head CT/CTA	Periventricular leukoaraiosis; global cerebral atrophy.	Age-related changes; no acute intracranial event.
Cervical CT	Multilevel disco-osteophytic disease; narrow canal.	Chronic degenerative spondylosis; SCIWORET risk factor.
Cervical MRI	T2/STIR hypersignal (bulbomedullary to C3); "H-sign".	Acute Spinal Cord Infarction secondary to low-flow.
CT TAP	RLL opacification; 2.5 cm hepatic nodule.	Aspiration Pneumonitis; incidental liver lesion.
Clinical Exam	Spastic tetraparesis (Grade 1/5); plegia of upper limbs.	SCIWORET; requires intensive neuro-rehabilitation.

CSF studies were unremarkable, with normal cell count, glucose, and protein. Autoimmune markers (antinuclear antibody (ANA), extractable nuclear antigen (ENA), aquaporin‑4 antibody) were negative. Electroencephalogram (EEG) and brain MRI excluded cortical involvement. An incidental hepatic nodule identified on CT was characterised as compatible with a benign haemangioma, remaining stable on follow-up imaging performed a year later. 

The patient was managed with supportive ICU care, prioritizing maintenance of a mean arterial pressure (MAP) above 70 mmHg to preserve spinal cord perfusion pressure, achieved during the ICU stay without vasopressor support. High-dose methylprednisolone was discussed with Orthopaedics and Neurology; both specialties advised against its administration, citing the absence of evidence supporting corticosteroid use in non-traumatic spinal cord infarction, particularly beyond the 72-hour window. Cardiology was consulted regarding the important troponin elevation and concluded that, in the context of type 2 myocardial infarction secondary to systemic hypoperfusion, there was no indication for therapeutic anticoagulation or antiplatelet therapy.

After three days of mechanical ventilation, the patient was successfully extubated. Physiotherapy and passive limb mobilization started on day 4. The patient remained hemodynamically stable throughout his ICU stay, without secondary infections or renal dysfunction. The colonoscopy was ultimately not performed, as gastrointestinal symptoms resolved completely upon clozapine discontinuation, consistent with clozapine-induced ischaemic colitis. He was transferred to the Internal Medicine ward and subsequently admitted to a specialized intensive rehabilitation facility. Over several months of multidisciplinary therapy, he experienced a remarkable recovery. At final follow-up, his gait was restored, and he was almost fully autonomous, with only a 4/5 strength deficit in his left side body, and intermittent sphincter incontinence.

## Discussion

The pathophysiology of SCIWORET in this adult patient suggests a synergistic "triple-hit" mechanism involving metabolic, hemodynamic, and mechanical insults.

The first hit was the metabolic crisis. Severe hyponatremia (Na+ 115-118 mmol/L) is a well-documented complication of bowel preparation, particularly in patients on psychotropic medications [5,6]. Severe hyponatremia may have triggered a seizure, acute encephalopathy, or a direct cardiac arrhythmia, any of which could have precipitated the respiratory collapse and subsequent cardiac arrest. In the absence of EEG data or witnessed seizure activity, the exact triggering mechanism remains speculative; however, the temporal association with the hyponatremic state and the absence of primary cardiac ischaemia on subsequent workup support hyponatremia as the principal precipitant. Furthermore, it is hypothesised that hyponatremia-induced cellular edema may have further compromised microvascular perfusion pressure within the already stenotic spinal canal, though this mechanism has not been formally demonstrated in the spinal cord ischaemia literature and should be regarded as speculative [5].

The second hit was the hemodynamic low-flow state. During the cardiac arrest, the patient experienced systemic hypotension. The spinal cord gray matter, specifically the anterior horns, possesses a high metabolic demand and is acutely sensitive to ischemia [7]. The "H-sign" (or "owl's eye" sign) seen on MRI confirms that the ischemic insult was localized to the territory of the anterior spinal artery, which supplies the anterior two-thirds of the spinal cord, including the metabolically demanding anterior horns. This territory represents a watershed area highly vulnerable to systemic hypoperfusion. In this case, the approximately two-minute cardiac arrest resulted in a profound drop in MAP, providing an unequivocal hemodynamic mechanism for this localized spinal cord infarction [8,9].

The third hit was the mechanical predisposition. The MRI confirmed a narrow spinal canal and multilevel spondylosis. Chronic cord compression likely compromised the microvascular collateral circulation, acting as a locus minoris resistentiae [2,4]. In a healthy spine, the cord might have survived a brief period of low flow; however, in this stenotic canal, the microvascular reserve was already exhausted.

This report has limitations inherent to its retrospective, single-case design. Formal structured outcome measures such as the American Spinal Injury Association (ASIA) Impairment Scale were not prospectively recorded, limiting direct comparison with published SCIWORET series. Future prospective registries should incorporate standardised neurological and functional assessments to better characterise outcomes in this population.

The presentation and outcome of this case are contextualized by comparison with published spinal cord infarction series. Ros Castelló et al. described 41 patients with spinal cord infarction, of whom 58.5% were male with a mean age of 61 years; motor deficits were present in 95.1%, sensory deficits in 80.4%, and autonomic dysfunction in 58.5% [10]. The clinical profile of our patient, a 67-year-old male with tetraparesis, hypesthesia, and sphincter incontinence, is consistent with these series characteristics. Regarding prognosis, published series consistently report poor long-term functional outcomes, with complete or near-complete recovery achieved in a minority of cases [7,8,10]. The near-total functional recovery in our patient therefore represents a notably favourable outcome, likely attributable to the brevity of the ischaemic insult, the absence of aortic or embolic pathology, and the intensity of early specialist rehabilitation. In the systematic review by Dokponou et al., the owl's eye sign was most frequently reported at the cervical level (39.6%), consistent with the axial MRI findings in the present case [8].

This case emphasizes that SCIWORET must be considered in the differential diagnosis of any post-arrest patient with persistent motor deficits. The assumption that motor weakness is always "brain-related" in post-arrest patients can lead to significant diagnostic delays. The excellent functional outcome in this patient, achieving near-total recovery after specialized intervention, underscores the importance of early diagnosis and the transfer to high-intensity rehabilitation centers [10].

## Conclusions

This case highlights the importance of considering SCIWORET in post-arrest patients presenting with neurological mismatch. Severe metabolic derangements, such as bowel-preparation-related hyponatremia in high-risk patients, may synergistically contribute to spinal cord ischaemia through a 'triple-hit' mechanism. When neurological mismatch is identified, urgent spinal MRI should be performed to exclude focal spinal cord pathology before attributing deficits solely to hypoxic-ischaemic encephalopathy. The "H-sign" on axial T2-weighted images is a radiological hallmark of anterior spinal artery infarction and should be actively sought in this context. Early specialist rehabilitation is strongly supported by the outcome achieved in this patient, who attained restored gait and near-full autonomy, despite residual left-sided 4/5 strength deficit and intermittent sphincter incontinence, a remarkably favourable outcome.
